# Analysis of Binding Interactions of Ramipril and Quercetin on Human Serum Albumin: A Novel Method in Affinity Evaluation

**DOI:** 10.3390/molecules25030547

**Published:** 2020-01-27

**Authors:** Zuzana Vaneková, Lukáš Hubčík, José Luis Toca-Herrera, Paul Georg Furtműller, Pavel Mučaji, Milan Nagy

**Affiliations:** 1 Department of Pharmacognosy and Botany, Faculty of Pharmacy, Comenius University in Bratislava, 83232 Bratislava, Slovakia; 2 Department of Physical Chemistry of Drugs, Faculty of Pharmacy, Comenius University in Bratislava, 83232 Bratislava, Slovakia; 3 Institute of Biophysics, Department of Nanobiotechnology, University of Natural Resources and Life Sciences, 1180 Vienna, Austria; 4 Institute of Biochemistry, Department of Chemistry, University of Natural Resources and Life Sciences, 1180 Vienna, Austria

**Keywords:** human serum albumin, ramipril, quercetin, fluorescence, microscale thermophoresis, circular dichroism, molecular docking

## Abstract

The aim of this study was to analyze the binding interactions between a common antihypertensive drug (ramipril, R) and the widely distributed plant flavonoid quercetin (Q), in the presence of human serum albumin (HSA). From the observed fluorescence spectra of the (HSA + R) system we can assume that ramipril is also one of the Site 3 ligands—similar to fusidic acid—the binding of which has been proven by RTG crystallography. Our claim is supported by near-UV CD spectroscopy, microscale themophoresis and molecular modeling. The presence of R slightly inhibited the subsequent binding of Q to HSA and, on the contrary, the pre-incubation of HSA with Q caused a stronger binding of R, most likely due to allosteric interactions. At high concentrations, R is also able to displace Q from its binding site. The dissociation constant K_D_ for the binding of R is more than hundredfold larger than for Q, which means that R is a very weak binder to HSA. The knowledge of qualitative and quantitative parameters of R, as well as the methods used in this study, are important for future research into HSA binding. This study shows the importance of implementing other methods for K_D_ determination. Microscale thermophoresis has proved to be a novel, practical and accurate method for K_D_ determination on HSA, especially in cases when fluorescence spectroscopy is unable to produce usable results.

## 1. Introduction

Human serum albumin (HSA) is the most common protein in human plasma, constituting around 60% of the total plasma proteins and serving mainly as a transporter [1]. HSA is able to bind many different types of compounds (e.g., ions, fatty acids, bile acids, drugs and their metabolites, etc.). There are several well-researched binding sites on HSA: Sudlow’s Site 1 (subdomain IIA, a large multichamber pocket defined mainly by the single tryptophan residue) and Sudlow’s Site 2 (subdomain IIIA, shaped similarly to Site 1, but the compounds bound here usually contain a peripherally located negative charge) [2,3].

Recently there has also been a number of studies published regarding the third main drug-binding site located on subdomain IB. It is the primary binding site of bilirubin, hemin, a sulphonamide derivative, and the steroid antibiotic fusidic acid [4,5,6]. This binding site is characterized by two tyrosine residues (Tyr^138^ and Tyr^161^) and circular dichroism appears to be a useful method for detection of the binding of potential ligands [4,7].

In the presence of multiple ligands, HSA creates a complex system with several possible binding combinations and outcomes. The ligands might compete for one of the binding sites, there may exist an allosteric interaction where a ligand bound to one binding site influences another binding site’s ability to bind a different ligand. The importance of the interaction studies lies within the clinical importance of some cases, since only the free unbound fraction of the drug carries the therapeutic effect. Bilirubin displaced from its binding site by fusidic acid or sulphonamide antibiotics can cause neonatal jaundice [5], warfarin can be displaced by several Site 1 drugs, causing irregularities in blood clotting [3,8]. Even some plant compounds may cause such interactions, regardless of whether they are coming from dietary sources or from food supplements or pharmaceutical preparations.

**Figure 1:**
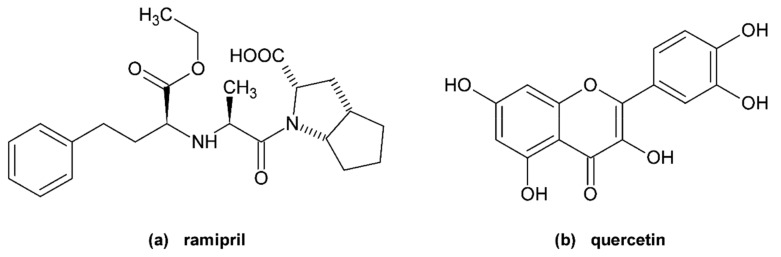
Structures of (**a**) ramipril and (**b**) quercetin.

Ramipril (R, Figure 1a) is a prodrug which is hydrolysed after absorption by carboxylesterase 1 to form the active angiotensin converting enzyme (ACE) inhibitor, ramiprilat. Ramiprilat decreases the plasma levels of angiotensin II and aldosterone and potentiates the effects of bradykinin. Over the concentration range of 0.01 to 10 mg/L, ramipril and ramiprilat are 73% and 56% bound, respectively, to human serum proteins when measured by equilibrium dialysis [9]. According to Shi et al. [10], ramipril binds to Site 1 of bovine serum albumin (BSA) with dissociation constant K_D_ = 28.5 μM. It is, however, yet to be seen if this result can be applied to HSA. The patents and patent applications of Carter et al. suggest that ramipril binds to subdomain IB but these claims have not been confirmed by any additional study [4].

Quercetin (Q, Figure 1b) is a commonly occurring flavonol. It is a strong antioxidant with additional anti-inflammatory and venoprotective properties. A regular healthy diet contains 25–50 mg of Q daily. Q and its metabolites (mainly quercetin glucuronide) bind strongly to HSA (99% for Q) [11,12], the main binding site is Sudlow’s Site 1 with a K_D_ previously determined by our research team to be 6.1 μM [13] and 6.48 μM [14].

The aim of this study was to determine the binding of the above-mentioned two compounds to HSA and investigate the possibility of mutual binding interactions. This drug combination has, to the best of our knowledge, not yet been studied. Moreover, in the case of ramipril, there is an insufficient amount of information about its binding behavior. We utilized the standard methods used in HSA interaction research: fluorescence spectroscopy and circular dichroism. These methods were supplemented by a current method—Microscale thermophoresis (MST). Additionally, a molecular docking was implemented to visualize the suggested results.

## 2. Results and Discussion

### 2.1. Fluorescence Spectroscopy

Intrinsic fluorescence of proteins is caused by aromatic amino acid residues, i.e., tryptophan (Trp), tyrosine (Tyr), and phenylalanine (Phe). Tryptophan fluorescence emission is usually dominant in the protein spectrum, with an excitation maximum wavelength at 295 nm and an emission maximum wavelength at 350 nm. Tyrosine emissions occur at shorter wavelengths and are usually quenched by the surrounding protein matrix; therefore, they are not commonly utilized for binding research.

Any change of the micro-environment around these fluorescent amino acid residues results in a change of the emission spectra. These include changes in the solvent and protein matrix polarity, folding and unfolding of the protein backbone, complex formation, weak bonds or collisional quenching. They result in changes of the fluorescence intensity (quenching or enhancement) and shifts in the maximum wavelengths—blue shift towards shorter wavelength and red shift towards longer wavelength [15].

Although there is not any significant difference between HSA and BSA when it comes to their function (they both exist mainly to bind and transfer small molecules and fatty acids through the bloodstream), there are some differences when it comes to the comparison of results obtained on serum albumins of different species. It has been proven that the results obtained on BSA should not be extrapolated to HSA without at least a few confirmatory measurements [16]. Warfarin, a commonly used golden standard for albumin binding measurements, gives varied results when bound to HSA and BSA, and these results are further divided by the different reactions of these albumins to chosen buffers [17]. In other cases, the binding sites for the same molecule can be completely different on HSA and BSA, as HSA Site I is more malleable and offers more structural adaptability than BSA, which seems to be very rigid in comparison [18].

Most importantly, BSA contains two Trp residues (as opposed to only one in HSA). This second tryptophan (Trp^134^) is located on subdomain IB, close to the third binding site.

**Figure 2:**
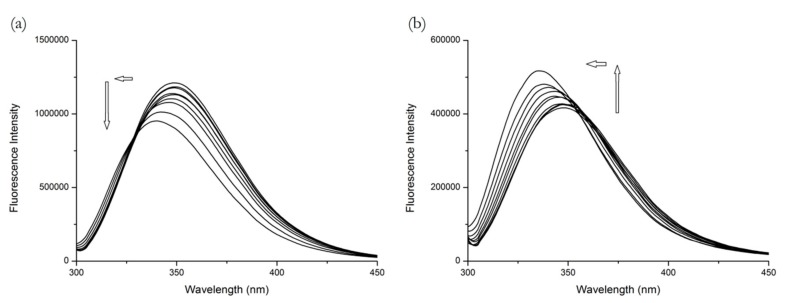
The fluorescence emission spectra of (**a**) BSA + R and (**b**) HSA + R systems at λ_EX_ = 295 nm. Conditions: T = 310.15 K, pH = 7.4. The human serum albumin (HSA) and bovine serum albumin (BSA) concentrations were 5 μM; R concentrations were 15.6, 31.25, 62.5, 125, 250, 500, 1000, 2000 and 4000 μM.

From Figure 2, we can see that ramipril is also an example of when the results from BSA cannot be directly extrapolated to HSA. On the BSA figure, we can see the fluorescence intensity is decreasing with the increasing concentration of R, which is consistent with the findings of Shi et al. [10] and could suggest binding to Site 1 on the first glance. However, on the HSA figure, the increasing concentrations of R cause a fluorescence intensity increase. In both cases the changes were accompanied by a remarkable blue shift, which means that the polarity around the Trp^214^ residue decreased. The extremely high concentration of R necessary to achieve even a small change of fluorescence suggests that R is a very weak binder.

This dualistic behavior, unusual in common binding studies, might be explained using the studies by Zhdanova et al. [19,20] as an analogy. As the R concentration increased the Trp^214^ fluorescence of analogous proteins exhibited different behavior. The increase in Trp^214^ (located in subdomain IIA of HSA) fluorescence observed by us could be due to a reduction in static quenching by neighboring amino acid residues taking place in the native protein structure. This can happen due to allosteric modulation of the subdomain IIA. The same removal of static quenching also occurs for Trp^213^ in BSA as its local environment is similar to that of Trp^214^ in HSA. Nevertheless, since we observe a decrease in BSA fluorescence, it signifies that it is a consequence of the binding interaction of ramipril on the other Trp residue, namely Trp^134^ located in the subdomain IB and characterized by the larger absorption cross-section and larger fluorescence lifetime, thus making a larger contribution to fluorescence intensity [17]. Thus, we suggest that ramipril is a Site 3 binder.

To further support this theory, we selected a known Site 3 binding molecule, fusidic acid (FA), for comparison. FA has been shown to bind strongly to HSA, displace bilirubin from its binding site on subdomain IB [5], and its binding site has been confirmed by X-ray crystallography [6].

**Figure 3:**
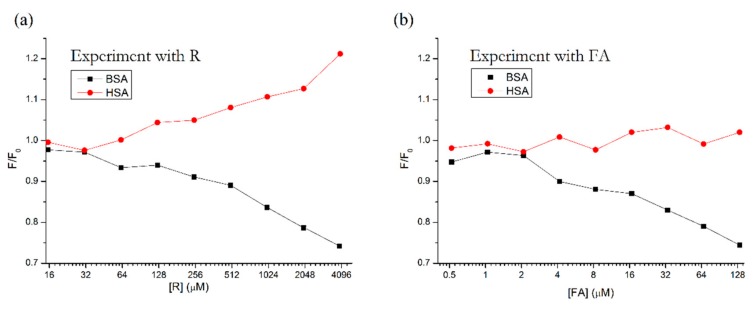
Side-by-side comparison of fluorescence intensity maxima of HSA + R and BSA + R (**a**) vs. HSA + FA and BSA + FA (**b**), according to the procedure described by Zhdanova et al., 2015 [20]. Conditions: λ_EX_ = 295 nm, T = 310.15 K, pH = 7.4. The HSA and BSA concentrations were 5 μM; FA concentrations were 0.52, 1.04, 2.08, 4.16, 8.33, 16.66, 33.33, 66.66 and 133.33 μM. R concentrations were 15.6, 31.25, 62.5, 125, 250, 500, 1000, 2000 and 4000 μM.

In Figure 3, we can see the similar behavior of these two compounds. Both R and FA cause the fluorescence of BSA to decrease, while the fluorescence of HSA either increases (R) or remains more or less constant (FA). Based on these results, we can assume that R also binds to the third binding site on subdomain IB.

**Figure 4:**
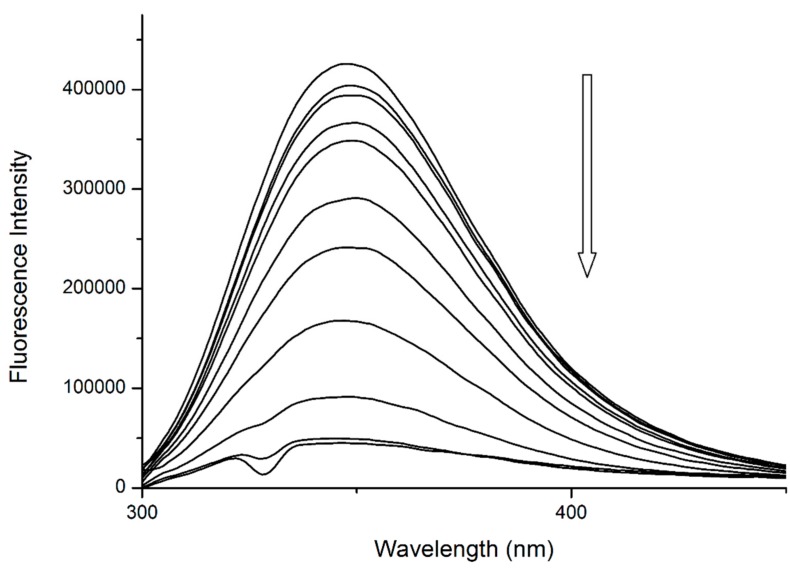
The fluorescence emission spectra of HSA + quercetin (Q) system at λ_EX_ = 295 nm. Conditions: T = 310.15 K, pH = 7.4. The concentration of HSA was 5 μM, the concentrations of Q were 0.12, 0.24, 0.49, 0.97, 1.95, 3.9, 7.8, 15.6, 31.25 and 62.5 μM.

The binding of quercetin has been already studied in detail. Previous publications by our research team confirmed that Q binds to the close proximity of Trp^214^, then quenches its fluorescence by static quenching (Figure 4) and forms a fluorescent complex with Trp^214^ [13,14]. 

To find out if there was any interaction when both studied compounds were present, we performed an analysis of ternary mixtures, [(HSA + Q) + R] and [(HSA + R) + Q], when the first-added compound was pre-incubated with HSA at a constant concentration and the second compound was added at variable concentrations. The concentrations of the first-added drug in the ternary system was 5 μM for Q and 400 μM for R. These concentrations were chosen as an approximate half-saturation point for both compounds.

The comparison of the quenching/enhancement curves allows us to see if the presence of another compound changed the affinity of the second binding partner.

**Figure 5:**
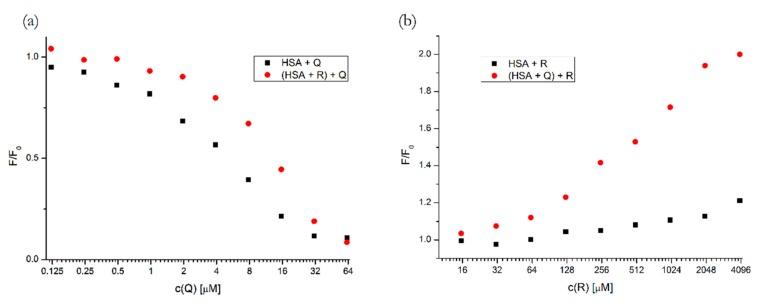
Quenching/enhancement curves of HSA with Q (**a**) and with R (**b**), respectively, for binary (black squares) and ternary (red dots) systems. Conditions: T = 310.15 K, pH = 7.4, λ_EX_ = 295 nm. The concentration of HSA was 5 μM, the concentrations of Q and R were increasing from 0.12 to 62.5 μM and from 15.6 to 4000 μM, respectively. The concentrations of the firstly added drug in the ternary system was 5 μM for Q and 400 μM for R.

In Figure 5, we can see that the presence of R slightly inhibited the binding of Q to HSA, since the quenching curve shifted towards higher concentrations.

Contrastingly, the pre-incubation of HSA with Q caused a more intense fluorescence enhancement by R. This can be explained by two different processes: (1) the binding of R could be increased due to the allosteric modulation of Subdomain IB by the previously bound Q; (2) as Q causes a slight Trp^214^ fluorescence quenching in the pre-incubated HSA, the subsequent binding of R might displace Q from its binding site, which would consequently reduce the quenching.

**Figure 6:**
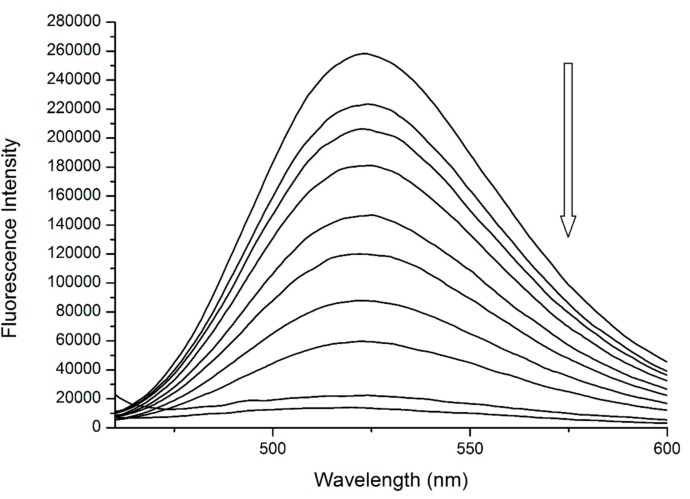
The fluorescence emission spectra of ternary system (HSA + Q) + R at λ_EX_ = 450 nm. Conditions: T = 310.15 K, pH = 7.4. The concentrations of HSA and Q were 5 μM in all samples, the concentrations of R were increasing from 15.6 to 4000 μM, respectively.

We can support this second assumption by analyzing the spectrum of Q + Trp fluorescent complex (Figure 6). This ground-state complex can be observed at λ_EX_ = 450 nm, with a maximum of λ_EM_ = 525 nm [14]. Upon the addition of R into the pre-incubated mixture of HSA + Q we can see that the band characteristic for bound Q is decreasing in intensity until it almost vanishes at the highest concentrations of R. One reason for this behavior is that the fluorescent complex was being disrupted and Q was being displaced from its binding site in Subdomain IIA—most likely due to allosteric modulation. Another possibility is that upon binding, R interacts with the Q + Trp fluorescent complex directly, causing the fluorescence quenching without any displacement of Q.

### 2.2. Stern–Volmer Analysis

Fluorescence quenching can be described by the well-known Stern–Volmer equation:

F_0_/F = 1 + K_q_ [Q] (1)

where F_0_ and F are the fluorescence intensities before and after a quencher addition, K_q_ is the Stern–Volmer quenching constant and [Q] is the quencher’s concentration [15]. The slope of the graph is therefore an indicator of the quencher’s behavior in the fluorophore’s proximity.

**Figure 7:**
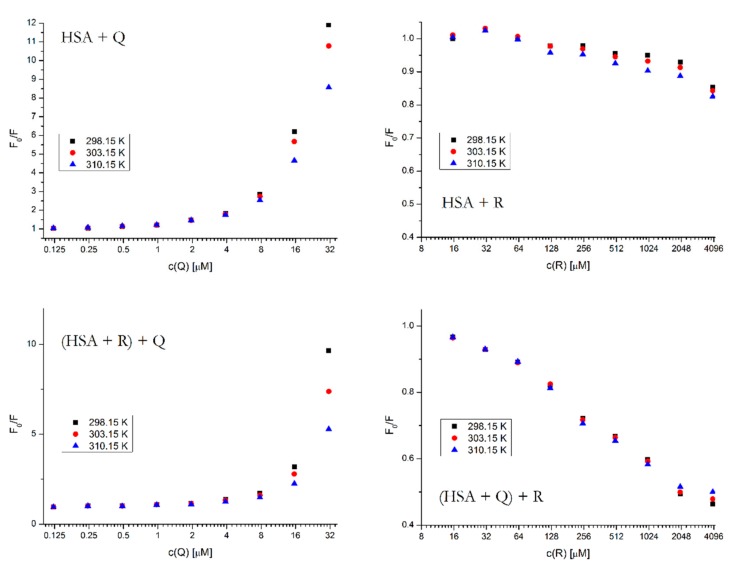
Stern–Volmer plots for all binary and ternary systems at different temperatures and λ_EX_ = 295 nm. The HSA concentration was 5 μM; the concentrations of Q and R were increasing from 0.12 to 62.5 μM and from 15.6 to 4000 μM, respectively. The concentrations of the firstly added drug in the ternary system was 5 μM for Q and 400 μM for R.

It is possible to determine the type of fluorescence quenching by comparing the K_q_ value at various temperatures. With the HSA + Q system, in Figure 7 we can see that the K_q_ value significantly decreases with the increasing temperature, which is typical for a fluorophore–ligand complex formation that gets disrupted if the system receives more energy in the form of heat [15]. The same effect is also observable in the ternary system [(HSA + R) + Q].

For the binding of R, we cannot be talking about quenching constants as such, since this compound causes an enhancement of fluorescence in HSA rather than quenching, therefore the Stern–Volmer equation is not applicable. However, the graph of F_0_/F vs. [R] is nevertheless able to give us the information about the strength of the binding at different temperatures. In the binary system HSA + R we can observe that the fluorescence enhancement is more intense at higher temperatures, which suggests that the binding of R does not involve a complex formation and it is stronger at higher temperatures, as the small molecule has more kinetic energy to wedge itself into the binding site. The ternary system is not as unambiguous, we see that most concentration points behave in a similar way (the binding being stronger at higher temperatures), only at the two highest concentration points is the progression reversed. This might imply that a more complicated process is occurring which involves both complex formation and collisional interactions of all binding partners.

### 2.3. Binding Constant Analysis

As previously stated, linear expressions of binding data (i.e., Scatchard plot) are not suitable nor accurate enough to determine an accurate K_D_ value [14]. Therefore, as in previously published research [14], we used the DynaFit software script. Data input consisted of [L_total_] (total ligand concentrations) for the variable reactant concentrations, [P_total_] (total protein concentration) for the fixed reactant concentration, and F/F_0_ for the experimentally observed value. The software calculates [L_free_] (free ligand concentration), creates a semilogarithmic plot of F/F_0_ vs. [L_free_] and determines the K_D_ value by finding the halfway point in the sigmoid-shaped curve.

**Figure 8:**
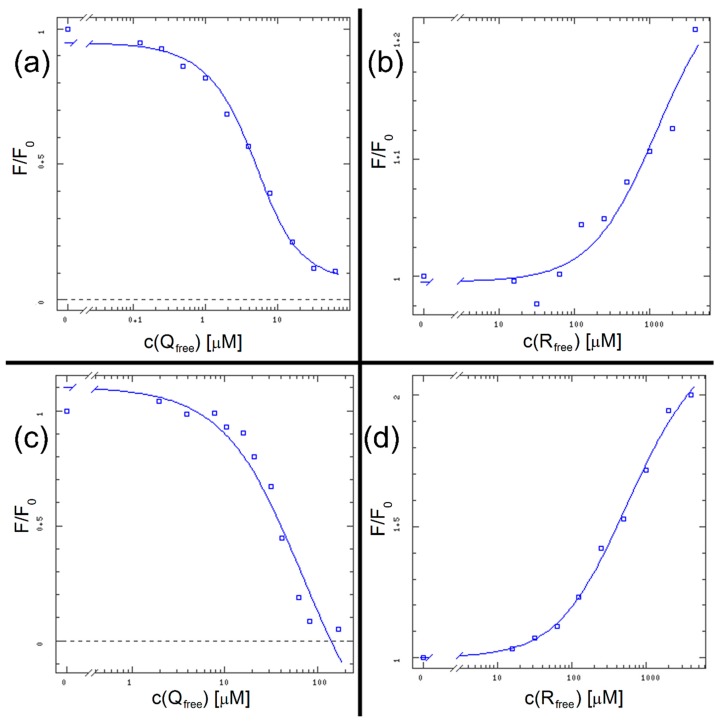
The binding curves for all systems: HSA + Q (**a**), HSA + R (**b**), (HSA + R) + Q (**c**) and (HSA + Q) + R (**d**).

The results are shown in Figure 8 and Table 1, along with the K_D_ results from the following method for direct comparison. We can see that in case of HSA + R binary mixture we did not approach the saturation point of the binding curve, therefore the K_D_ value determined by DynaFit comes with a significant error margin.

### 2.4. Microscale Thermophoresis

Ramipril, being a weak non-Site-1 binder, does not allow us to use fluorescence spectroscopy to accurately determine K_D_ values, since we were not able to reach the saturation point. Therefore, it was necessary to search for another method which would allow us to quantify the binding of R.

Microscale thermophoresis (MST) is a current method which allows us to determine the binding parameters in a rapid, accurate fashion using only minute amounts of sample solutions. It is based on thermophoresis, the directed movement of molecules in a temperature gradient, which strongly depends on a variety of molecular properties such as size, charge, hydration shell or conformation. Thus, this technique is highly sensitive to virtually any change in molecular properties, allowing for a precise quantification of molecular events independent of the size or nature of the investigated specimen.

During an MST experiment, a temperature gradient is induced by an infrared laser. The directed movement of molecules through the temperature gradient is detected and quantified using either covalently attached or intrinsic fluorophores [21].

**Figure 9:**
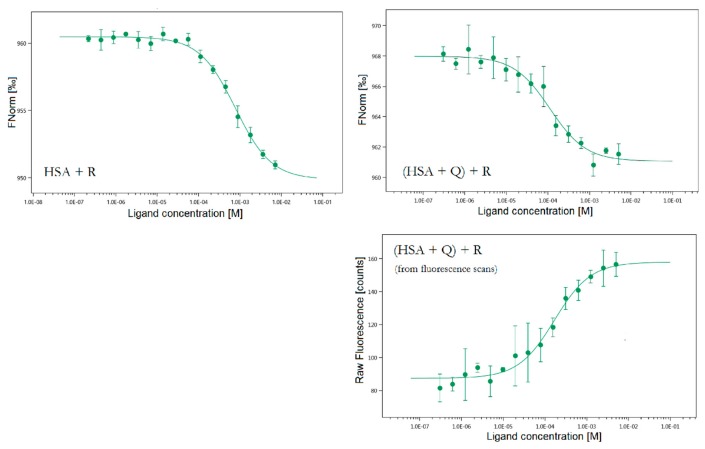
The MST binding curves for all studied systems. Conditions: T = 297.05 K, pH = 7.4. The concentration of HSA was 20 nM, the starting concentrations of R were 7.15 mM, c(Q) in the ternary system was 20 nM.

We performed a full set of MST measurements for mixtures where R was the compound with the concentration gradient. The results are displayed in Figure 9 and Table 1.

An interesting observation regarding HSA + R system was that the results displayed in Table 1 were automatically obtained at an early timepoint during the experiment (second 1 out of approx. 20). If we selected a later timepoint, the K_D_ values for R got progressively lower with the increasing temperature in the capillaries. That could suggest that the binding of R is stronger at higher temperatures, which is in accordance with the Stern–Volmer analysis. The K_D_ value obtained here is one order of magnitude lower than the one from fluorescence spectroscopy but considering the high error margin in this system’s fluorescence binding curve, the MST result appears valid and more accurate.

For [(HSA + Q) + R] system it was possible to additionally derive K_D_ value from the increase in fluorescence in the initial capillary scans. The mechanism of this feature is the same as in the regular fluorescence experiment (Figure 5). For this system, all K_D_ values from three separate methods are in hundreds of μM which confirms the result and shows that MST is a suitable and precise method for HSA binding studies.

### 2.5. Circular Dichroism Spectroscopy

Circular dichroism spectroscopy is a valuable technique for detecting changes in the protein secondary and tertiary structure, using detections of changes in the total ellipticity. Since the proteins are usually comprised of chiral amino acid residues, this method is one of the most sensitive towards tiny changes in the protein structure and micro-environment. The major downside lies in the immense complexity and variability of proteins, and also in the large number of amino acid residues, therefore the evaluation is usually only empiric [22].

In order to obtain an insight into the HSA structure, the far-UV (200–260 nm) and the near-UV (250–340 nm) CD spectra were recorded in the presence or absence of drugs in six molar ratios of ligands to protein (L:P) (0, 0.5, 1, 2, 3, and 4), for both binary and ternary systems.

**Figure 10:**
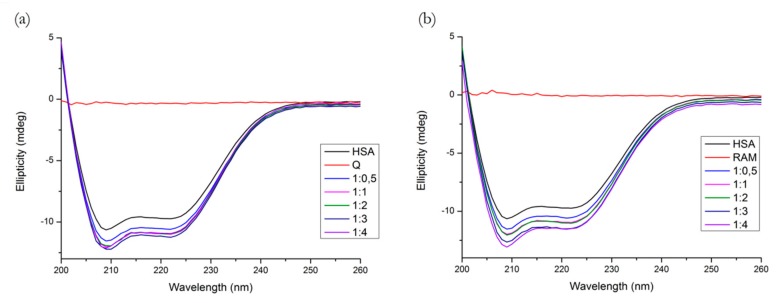
Far-UV CD spectra of (**a**) HSA + Q and (**b**) HSA + R systems. Conditions: T = 310.15 K, pH = 7.4. The HSA concentration was 1 μM; Q and R concentrations increased from 0 to 4 μM.

The far-UV CD spectrum of HSA showed two negative minima in the UV region at 208 nm and 222 nm, which is characteristic of the α-helical structure of HSA. The spectral profiles of the binary systems shown in Figure 10 indicate that the binding of both Q and R induced a minor perturbation in the HSA secondary structure.

Table 2 lists the α-helix percentage values for all molar ratios. Likewise, we see that the binding of both compounds significantly increased the α-helix percentages and induced changes in the protein secondary structure, R on a larger scale than Q.

**Figure 11:**
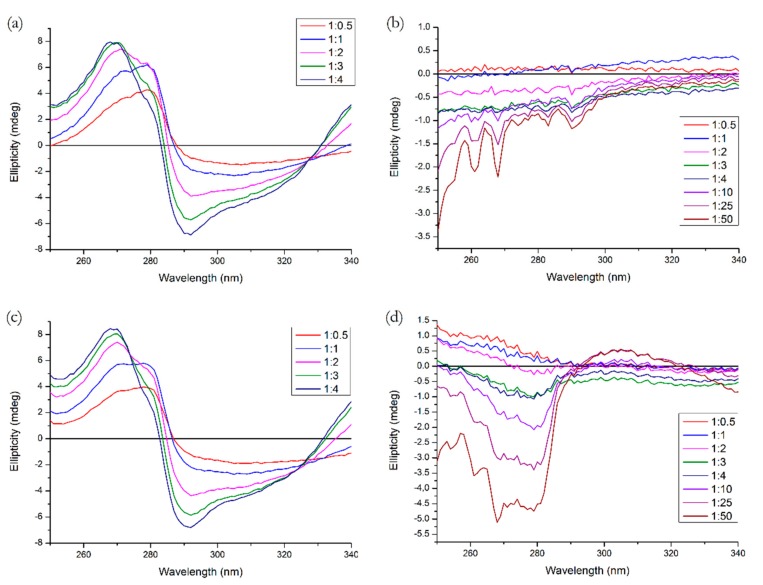
Near-UV CD spectra of (**a**) HSA + Q, (**b**) HSA + R, (**c**) (HSA + R) + Q, (**d**) (HSA + Q) + R. Conditions: T = 310.15 K, pH = 7.4. The HSA concentration was 15 μM, the ligand concentration ratios are listed in the legend.

The near-UV CD spectrum is a useful tool for observation of the protein tertiary structure. Figure 11 shows the spectra for all binary and ternary systems, with three additional ratios for R (10, 25 and 50 to 1, respectively). We can see that Q causes significant changes both in Tyr and Trp range (275–287 nm and 285–305 nm, respectively) [7], which is similar to the results of the study by Zsila et al. [23]. R, on the other hand, causes some minor changes in the Tyr range, which agrees with the fact that Site 3 on subdomain IB contains Tyr^138^ and Tyr^161^, which are sensitive towards conformational changes [4]. In a ternary system with HSA pre-incubated with R and variable Q concentrations, we do not see any particular difference compared to the binary system. On the contrary, pre-incubation with Q significantly increased the structural perturbations caused by the binding of R, which is in agreement with the fluorescence experiment. Q was able to enhance the binding of R in Tyr range and we see a positive band in the Trp range which might indicate the displacement of Q.

### 2.6. Molecular Modeling

**Figure 12:**
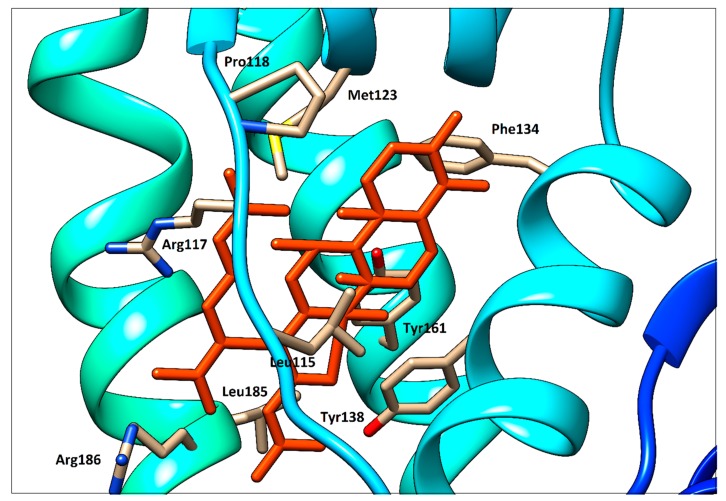
Detailed view of fusidic acid (orange red) docked with HSA.

**Figure 13:**
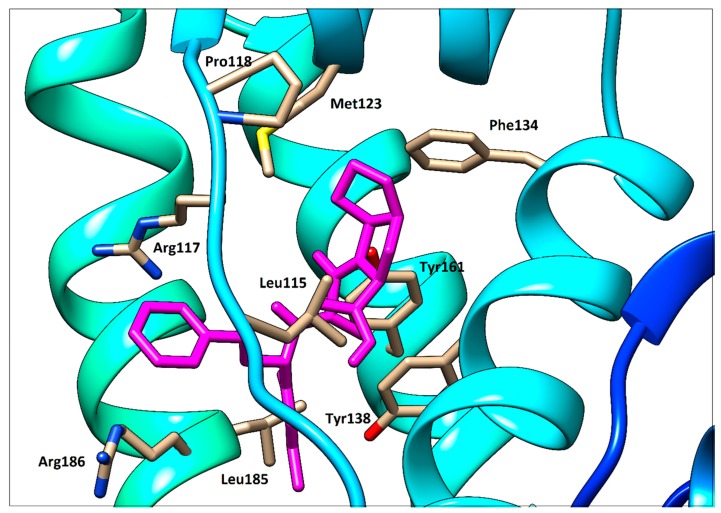
Detailed view of ramipril (magenta) docked with HSA.

**Figure 14:**
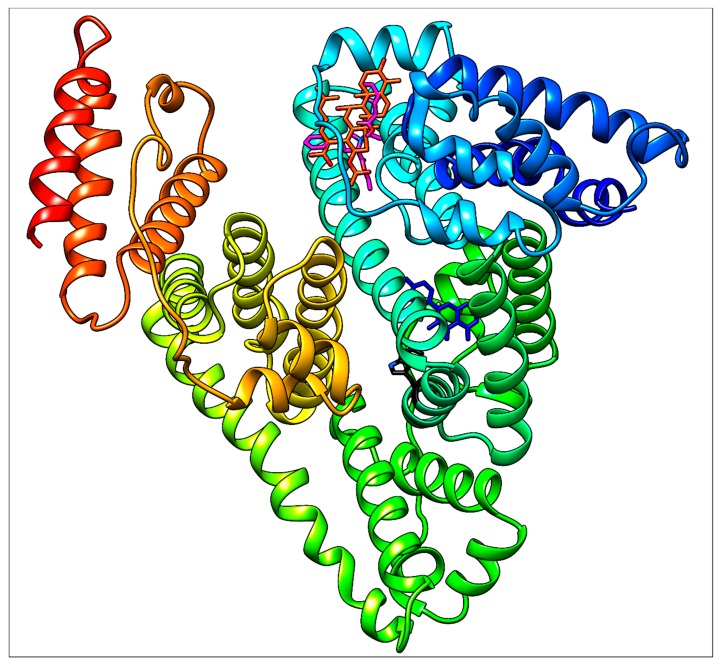
General view of HSA (with Trp^214^, black) docked (separately) with quercetin (blue), ramipril (magenta) and fusidic acid (orange red).

To support the interpretation of our spectroscopic results, quercetin, ramipril and fusidic acid were separately docked with HSA using PatchDock web service. As shown in our previous study [14], quercetin occupies binding site I (subdomain IIA, Figure 14). Fusidic acid occupies subdomain IB as previously demonstrated by X-ray diffraction with following amino acids in the ligand surroundings described: Leu^115^, Arg^117^, Pro^118^, Met^123,^ Phe^134^, Tyr^138^, Ile^142^, Phe^149^, Leu^154^, Phe^157^, Tyr^161^, Phe^165^, Arg^186^ and Gly^189^ [6]. An analogous situation was observed in our fusidic acid docking result, which confirmed a sufficient accuracy of the docking procedure used (Figure 12). Thus, the docking result for ramipril—again within subdomain IB—seems to be verified (Figure 13). However, contrary to the findings of [6], we could not find any hydrogen bonds between HSA and ramipril using the docking service approach, which is less accurate than the X-ray method.

Ramipril situated in subdomain IB is very far from Trp^214^ (Figure 14) which is responsible for the fluorescence quenching used routinely in HSA−ligand binding studies. This distance is approximately 20.5 Å, as determined using the UCSF Chimera 1.13 package. Due to this long distance, no quenching effect on the intrinsic fluorescence of HSA caused by ramipril (and fusidic acid, of course, too) can be observed.

## 3. Conclusions

The third drug binding site on HSA, located on subdomain IB, is one of the least described binding sites of HSA. There are only few securely confirmed small molecules that bind here, one of them being fusidic acid (FA). From the observed (HSA + R) system fluorescence spectra—and compared with the (HSA + FA) system—we can assume that ramipril is also one of the Site 3 ligands. This also appears to be the case with molecular modeling experiments, where R and FA were separately docked to subdomain IB, to the close proximity of two Tyr residues, Tyr^138^ and Tyr^161^. The same amino acid residues might be responsible for the near-UV CD spectrum changes, which were observed in the Tyr wavelength range.

The binding of Q to Sudlow’s Site 1 has been previously described in detail by our research team [13,14] as well as others. The interaction of Q with R seems to happen at higher concentrations of both small molecules: the presence of R slightly inhibited the following binding of Q to HSA and, on the contrary, the pre-incubation of HSA with Q caused a stronger binding of R, most likely due to allosteric interactions, since Q causes major changes in the protein’s tertiary structure, as can be seen on near-UV CD spectra. At high concentrations, R is also able to displace Q from its binding site.

The dissociation constant K_D_ for the binding of R is more than hundredfold larger than the one for Q, which means that R is a very weak binder to HSA. This data, combined with the fact that the concentrations necessary to achieve binding are essentially unattainable in vivo, means that the interaction between R and Q during plasmatic distribution appears to be clinically insignificant. It might be interesting to notice that R is commonly administered together with another antihypertensive—amlodipine—which has the potential of a clinically significant interaction with Q [14]. This drug combination has been produced as a fixed dose combination pill (Egiramlon^®^ and its generics); therefore, amlodipine’s interaction potential should be considered even in combination therapy.

Nevertheless, the knowledge of the qualitative and quantitative parameters of R, as well as the methods used in this study, are important for the future research of HSA binding. This study shows the importance of implementing other methods for K_D_ determination, as the commonly used linear transformation of the binding data has been proven inaccurate [14]. Microscale thermophoresis has been proved to be a practical and accurate method for K_D_ determination, especially in cases where fluorescence spectroscopy is unable to produce usable results.

## 4. Materials and Methods

### 4.1. Materials

Human serum albumin (recombinant, expressed in rice), ramipril (≥98%, HPLC), fusidic acid (pharmaceutical secondary standard), quercetin (≥95%, HPLC) and dimethylsulfoxide (DMSO; ≥ 99.5% (GC) plant cell culture tested) were purchased from Sigma–Aldrich (St. Louis, MO, USA). Phosphate buffer (50 mM, pH 7.4) was prepared from Na_2_HPO_4_ × 12 H_2_O and NaH_2_PO_4_ × 2 H_2_O (p.a., Centralchem, Banská Bystrica, Slovakia). HSA stock solutions were prepared by dissolving an appropriate amount in phosphate buffer. Quercetin, ramipril and fusidic acid stock solutions were prepared by dissolving the substance in DMSO and then diluting in phosphate buffer to the required concentration; the concentrations are listed in the figure descriptions of each experiment. DMSO concentration in final mixtures did not exceed 5% (*v/v*). Milli-Q water was used for all the measurements.

### 4.2. Methods

#### 4.2.1. Fluorescence Measurements

Fluorescence spectra were measured in triplicates on a FluoroMax 4 spectrofluorimeter (Horiba Jobin Yvon Scientific, Irvine, CA, USA), equipped with a 1.0 cm path length quartz cell. The slit widths for the excitation and emission were 3.0 nm for all measurements. The temperatures used for the measurements were 298.15 K, 303.15 K and 310.15 K, respectively. Samples were incubated for 2 min. Buffer background was subtracted from the raw spectra. Fluorescence intensities were corrected for the absorption of excitation light and re-absorption of emitted light to decrease the inner filter using the following relationship [13]:

(2) $$F_{cor}=F_{obs}\times 10^{(A_{ex}+A_{em)/2}}$$

where F_cor_ and F_obs_ are the corrected and observed fluorescence intensities, respectively. A_ex_ and A_em_ are the absorbance values of the experimental solution (including all ligands) at excitation and emission wavelengths, respectively. Corrected spectra were smoothed to minimize the influence of the signal noise.

#### 4.2.2. Binding Constant Analysis

Fluorescence spectral data after correction and smoothing were used to calculate the dissociation constant (K_D_) for all studied systems. For evaluation and logarithmic plot fitting we used DynaFit software (version 4.0, BioKin Ltd., Watertown, MA, USA, http://www.biokin.com/dynafit/index.html) using a custom-written script.

#### 4.2.3. UV Absorption Measurements

UV absorption spectra were performed on Infinite M200 Tecan (Männedorf, Switzerland) using Sarstedt TC Plate 96 Well, Standard, F. The temperature was 310.15 K. Plates were incubated for 2 min. Buffer background was subtracted from the raw spectra.

#### 4.2.4. Microscale Thermophoresis (MST) Measurements

The MST measurements were performed on Monolith NT.115 (NanoTemper Technologies, GmbH, Germany) instrument using Premium capillaries. HSA sample was labeled using RED-NHS protein labeling kit. Assay buffer was PBS with added 0.1% Pluronic F-127 as the surface agent. Ligand samples were dissolved in DMSO and then diluted with the assay buffer to the final concentration. Serial dilutions of the ligand were prepared, the protein was then added and the mixtures were incubated for 10 min at laboratory temperature. Each experiment was performed in three parallel measurements. The results were evaluated in the instrument software (MO.Control v1.6, NanoTemper Technologies, GmbH, Germany).

#### 4.2.5. Circular Dichroism (CD) Measurements

The isothermal wavelength scan studies of HSA in the absence or the presence of Q and/or R were carried out in triplicates using a Chirascan CD spectrophotometer equipped with a Peltier type temperature controller (Applied Photophysics Ltd., Leatherhead, UK). The instrument was flushed with nitrogen with a flow rate of 5 L per minute, the path length was 1 mm in the far-UV and 1 cm in the near-UV, spectral bandwidth was set to 1 nm, the scan time per point to 5 s and the temperature was set to 310.15 K. Buffer background was subtracted from the raw spectra.

For the far-ultraviolet (far-UV) CD spectra (200–260 nm) the HSA concentration was 1 μM. For the near-UV CD (250–340 nm) spectra a HSA concentration of 15 μM was used. Six molar ratios of ligands to protein (L/P) (0, 0.5, 1, 2, 3 and 4) were investigated for both binary and ternary systems.

The far-UV spectral data were used to calculate the α-helix percentage using the following equation [22]:

(3) $$\alpha \;helix\;\%=\frac{-MRE_{208\;nm}-\;MRE_{\beta -sheets}}{-MRE_{\alpha -helix}-MRE_{\beta -sheets}}\times 100$$

where MRE_208 nm_ is the mean residue ellipticity value of the sample at the excitation wavelength of 208 nm, MRE_α-helix_ is the standardized value for a protein with 100% content of α-helixes and it is equal to 33,000 and MRE_β-sheets_ is the standardized value for a protein with 100% content of β-sheets and it is equal to 4000.

The near-UV spectral data were evaluated by an empiric method described by Zsila et al. [4,7,23] The spectra were brought down to a common baseline by subtracting the spectrum of pure HSA.

#### 4.2.6. Docking Study

Human serum albumin structure from the Protein Data Bank (PDB ID: 1E78A) (http://www.wwpdb.org) was used for calculations. Quercetin, fusidic acid and ramipril structures were created in ACD/ChemSketch 12.01 software (www.acdlabs.com) and converted from a *.mol file format to a *.pdb one by OpenBabel 2.3.2 (http://openbabel.org/wiki/Windows_GUI) and used without any optimization. A PatchDock web server (http://bioinfo3d.cs.tau.ac.il/PatchDock/index.html) was used to dock (complex type: protein–small ligand, clustering RMSD = 4.0). Best 20 docking solutions were evaluated for both ligands. Molecular graphics images were produced using the UCSF Chimera 1.13 package (Resource for Biocomputing, Visualization, and Informatics at the University of California, San Francisco, CA, USA).

## Figures and Tables

**Table 1:** The K_D_ values for all systems obtained by fluorescence spectroscopy and MST.

System	K_D_ [μM] (Fluor.)	K_D_ [μM] (MST)	K_D_ [μM] (MST Fluorescence)
HSA + Q	2.28 ± 0.61	-	-
HSA + R	1220.86 ± 677.20	774.82 ± 75.28	-
[(HSA + R) + Q]	34.73 ± 15.08	-	-
[(HSA + Q) + R]	592.90 ± 90.63	111.92 ± 24.60	167.95 ± 29.84

**Table 2:** The values of α-helix percentages for binary systems HSA + Q and HSA + R.

System	Ratio Protein:ligand	α-Helix Content
HSA + Q	1:0	59.1 ± 1.0
	1:0.5	63.0 ± 2.0
	1:1	65.4 ± 0.5
	1:2	65.2 ± 0.9
	1:3	66.2 ± 0.7
	1:4	66.3 ± 1.5
HSA + R	1:0	59.1 ± 1.0
	1:0.5	63.0 ± 2.6
	1:1	65.0 ± 1.7
	1:2	65.1 ± 0.5
	1:3	68.2 ± 1.1
	1:4	70.2 ± 1.3
